# Neural responses to feedback information produced by self-generated or other-generated decision-making and their impairment in schizophrenia

**DOI:** 10.1371/journal.pone.0183792

**Published:** 2017-08-24

**Authors:** Atsuhito Toyomaki, Naoki Hashimoto, Yuki Kako, Harumitsu Murohashi, Ichiro Kusumi

**Affiliations:** 1 Department of Psychiatry, Graduate School of Medicine, Hokkaido University, Sapporo, Hokkaido, Japan; 2 Graduate School of Education, Hokkaido University, Sapporo, Hokkaido, Japan; Centro de Neurociencias de Cuba, CUBA

## Abstract

Several studies of self-monitoring dysfunction in schizophrenia have focused on the sense of agency to motor action using behavioral and psychophysiological techniques. So far, no study has ever tried to investigate whether the sense of agency or causal attribution for external events produced by self-generated decision-making is abnormal in schizophrenia. The purpose of this study was to investigate neural responses to feedback information produced by self-generated or other-generated decision-making in a multiplayer gambling task using even-related potentials and electroencephalogram synchronization. We found that the late positive component and theta/alpha synchronization were increased in response to feedback information in the self-decision condition in normal controls, but that these responses were significantly decreased in patients with schizophrenia. These neural activities thus reflect the self-reference effect that affects the cognitive appraisal of external events following decision-making and their impairment in schizophrenia.

## Introduction

Schizophrenia is a type of psychosis characterized by delusion and hallucination. Schneider's first rank symptoms explain these conditions in detail. Patients with schizophrenia have delusions of being controlled, wherein one experiences one's feelings, impulses, thoughts, or actions as not one's own, but as being imposed by some external force. Thought insertion is the delusion that one's thoughts are not really one's own, but are being placed into one's mind by an external force. These symptoms are thought to reflect dysfunction of the self-monitoring system.

Frith theorized that self-monitoring dysfunction is specific to schizophrenia [1]. Self-monitoring is an essential cognitive process functions in associated with planning, controlling, and anticipating the consequences of actions. It contributes to the evaluation of and comparisons between ongoing motor actions and goal representations. Sensory predictions enable comparisons between expected and actual outcomes of an action under consideration. The predictive model states that an efference copy (corollary discharge) of an outgoing motor command can be used to generate such a sensory prediction [2]. The efference copy is thought to be one of the most elementary mechanisms used to distinguish self-generated vs. externally generated sensory perceptual events. During motor execution, the efference copy signal generates a predicted state that permits the sensory consequences of a movement to be anticipated and used to attenuate the perceptions related to these sensations. In addition, this signal may be used to distinguish movements generated by oneself from those generated by an external source. If the predicted sensory consequences match the actual sensory consequences, the movement is labeled as one’s own. This process is referred to as a sense of agency, which is a self-directive or self-generated sensation accompanied by one’s own actions. However, if the predicted and actual sensory consequences are discordant, as when one’s arm is passively moved by someone else, the movement is labeled as externally generated. A dysfunctional predictive mechanism would lead to inappropriate predictions and cause the misattribution of self-generated actions as externally generated. Patients with schizophrenia can demonstrate just such difficulties when self-generated actions are experienced as being of alien origin. This leads to delusions of control or the misperception of self-generated speech as an auditory hallucination.

Several studies have investigated self-monitoring dysfunction in schizophrenia using behavioral and psychophysiological techniques. They have mainly focused on differences between the processes of sensation resulting from a self-generated action and those resulting from actions that are externally generated. Ford and colleagues have conducted a series of psychophysiological studies of auditory perceptual processes during talking and listening using event-related brain potentials (ERPs) [1–6]. They measured ERPs elicited by self-generated speech sounds when participants were speaking in real-time and listening to the same speech sounds in playback. They showed that in normal controls, the auditory N1 component is smaller during the talking condition than during the listening condition, but that this effect is not observed in patients with schizophrenia. These results indicate that the efference copy accompanying utterances in advance generates a predicted state in the auditory area and thereby dampens auditory perception, but that the efference copy does not play such a role in schizophrenia. Subsequent studies suggest that the functional connectivity between the frontal cortex and the temporal cortex contributes to this perceptual prediction based on the efference copy, and that this neural network is dysfunctional in schizophrenia. Several behavioral studies have investigated somatosensory processes of tactile stimulation self-produced by movement of the subject vs. those of external devices or other persons [7, 8]. Patients with schizophrenia have abnormalities in motor-induced suppression during the tactile stimulation self-produced condition. Recently, Ford and colleagues investigated neural activity during self-produced somatosensory stimulation using time-frequency analyses of electroencephalography (EEG) and ERP data [6]. Interestingly, a neural oscillation (phase synchronization of oscillations across trials) just before the motor execution of self-paced button presses was associated with subsequent tactile sensation reflected by somatosensory ERPs. These neural oscillations were abnormally reduced in patients with schizophrenia. The efference copy signal might thus be associated with the neural processes that occur in preparation of motor execution or decision-making. This may reflect a general forward modeling process that involves modalities other than sensation.

The above-described studies investigated processes immediately following the execution of actions. It is likely that abnormal sensory predictions affect not only bodily sensation, but also cognitive appraisal of environmental changes. However, no study has ever investigated whether the sense of agency or causal attribution of external events produced by self-generated decision-making is abnormal in schizophrenia. We thus examined neural activity underlying cognitive processes in response to external events involved in self-generated action. We used a simple gambling task wherein positive and negative feedback information was presented after decision-making. This task is suitable for the investigation of the neural substrates of cognitive appraisal in response to external events. A modified gambling task has been used in an interesting study wherein two individuals were differently engaged in decision-making and evaluation of feedback information [9]. In this study, EEG data were collected from participants who made decisions leading to monetary gain or loss during a self-decision condition. At the same time, the participants observed another individual’s performance and were subjected to the same monetary gain or loss outcomes in the other-decision condition. Thus, we compared cognitive processes in subjects during both self and other decision conditions using EEG. It is likely that some processes associated with sensory prediction during the self-decision condition affect cognitive appraisal of feedback information. We can thus assess neural processing associated with the sense of agency in response to external events (i.e., feedback information) presented shortly after decision-making using this gambling task wherein two individuals participate.

The purpose of this study was to investigate neural responses to feedback information produced by self-generated or other-generated decision-making. We compared ERP waveforms and EEG oscillations associated with the self-involvement effect between patients with schizophrenia and normal subjects during the performance of the above multiplayer gambling task.

## Methods

### Subjects

We obtained electrophysiological measurements from 11 patients with schizophrenia, who met the criteria for the disorder described in the Diagnostic and Statistical Manual of Mental Disorders, Fourth Edition (DSM-IV), and 11 normal subjects. Diagnosis of schizophrenia was made using a structured clinical interview for axis I disorders described in the DSM-IV. The mean age of the patients was 28.5 years (6 women and 5 men, aged 17–39 years, standard deviation [SD] = 7.8). The mean duration of illness was 7.3 years (SD = 7.7). The mean age of the normal subjects was 27.9 years (6 women and 5 men, aged 21–42 years, SD = 6.9). There was no significant difference in age between the patients with schizophrenia and the normal subjects (Student’s t-test, p > 0.6). All patients were taking atypical antipsychotics at the time of testing. Five patients were taking risperidone (mean chlorpromazine [CPZ] equivalent dose ± SD, 100.0 ± 0.0 mg/day), 3 patients were taking olanzapine (mean CPZ equivalent dose ± SD, 800.0 ± 0.0 mg/day), two patients were taking perospirone (mean CPZ equivalent dose ± SD, 300.0 ± 282.8 mg/day), and one patient was taking quetiapine (CPZ equivalent dose, 454.5 mg/day). Clinical symptoms were assessed using the Positive and Negative Syndrome Scale (PANSS) [10]. The mean values for positive, negative, and general psychopathology scale scores were 19.1 (SD = 9.7), 20.9 (SD = 6.7), and 39.3 (SD = 10.9), respectively. The local ethical committee of Hokkaido University approved this study. Written informed consent was obtained after a complete explanation of the study was provided to the patients. We obtained written consent from the guardians on behalf of children.

### Procedure

**Fig 1:**
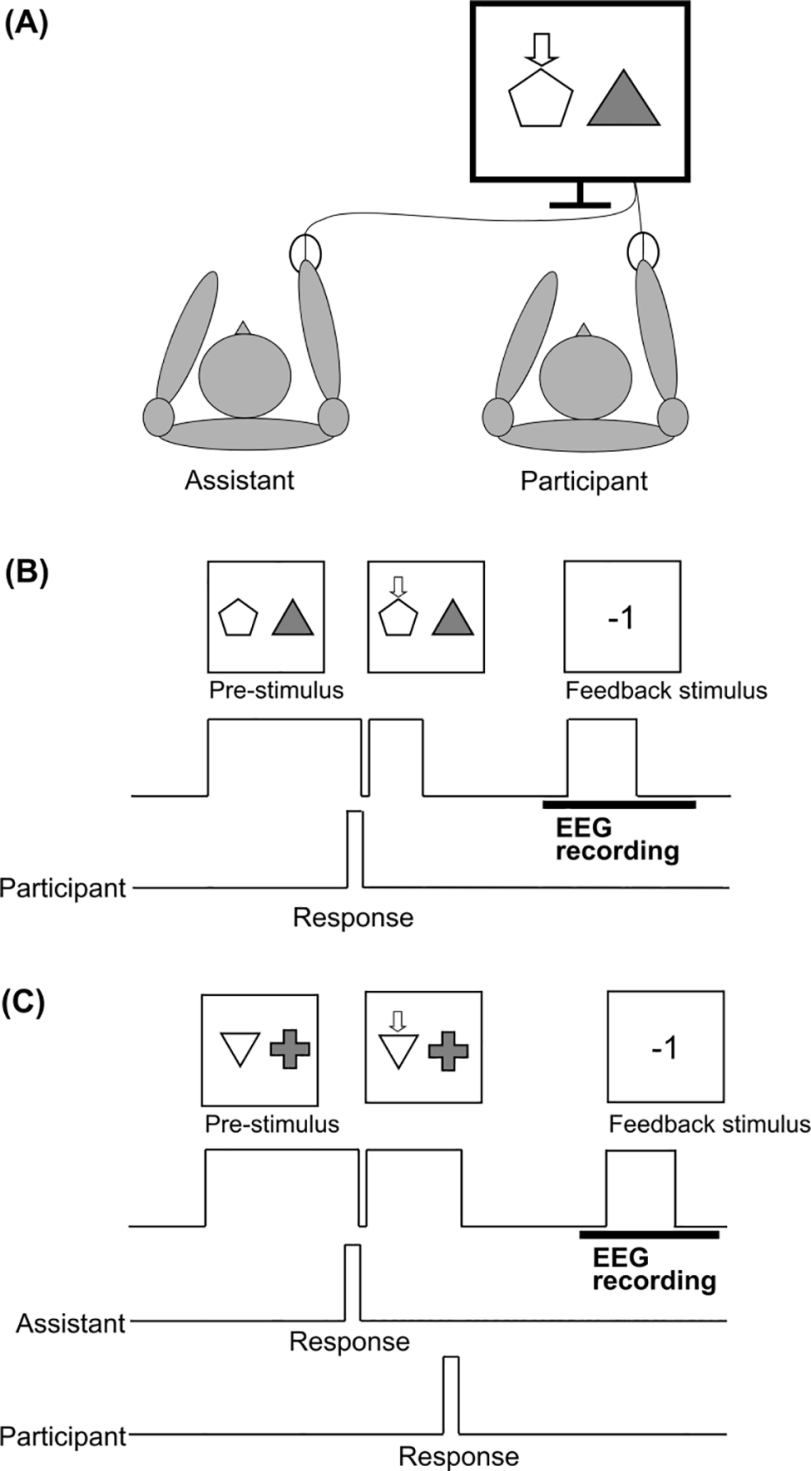
(A) Seating locations of the participant and the assistant. (B) Schematic diagram of the trial in the self-decision condition (C) and in the other-decision condition. The participant was seated in front of the display and instructed to select one of two figures, which were assigned gain or loss points, in the self-decision condition. The subjects were told to press the button according to the response selected by the assistant in the other-decision condition.

The participants were seated comfortably 1 m in front of a computer screen. An assistant sat beside the participant (Fig 1A). The participants performed a gambling task wherein the participants or the assistant had to choose one of two figures (left or right). This was followed by the presentation of feedback information (gain or loss). Each figure was assigned a probability of producing a “+1” (gain) or a “-1” (loss). We used two task conditions: the self-decision condition and the other-decision condition. Fig 1B and 1C show a schematic diagram of the gambling task used. In the self-decision condition, the participants had to choose one of two options and respond by pressing the mouse button. This was followed by the feedback stimulus (Fig 1B). The left figure was assigned a value of “+1” with a probability of 0.2 (“-1” with a probability of 0.8), and the right figure one was assigned a value of “+1” with a probability of 0.8 (“-1” with a probability of 0.2). The participants were not informed of this rule and were instructed to learn the rule and to gain as many points as possible. In the other-decision condition, the assistant had to choose one of two options and respond by pressing the mouse button. The participant then had to press the mouse button indicated on the computer screen (Fig 1C). In other words, the participants had to follow the assistant’s choice. As a result, the participants’ actions were made without their volition. The feedback stimulus was presented after the participant’s response. The two figures were assigned values of “+1” and “-1” at random, although we did not inform the participants of this fact. We told the participants that there is a rule, such as that used during the self-decision condition, and that they should determine this rule during the task. The assistant knew that points were assigned randomly in this condition, but was asked to choose each figure over several trials.

It is important to note that points were assigned to the participants during both the self and other decision conditions. We therefore instructed the participants to do their best to determine the rule used and to pay attention to whether they gain or lose points following the feedback stimuli during the experiment. The patterns of paired figures were changed for the self- and other-decision conditions so that the rule was different between the two conditions.

The pre-stimulus remained on the screen until the participant or the assistant pressed a mouse button. The participant and the assistant held their own mouse in their dominant hand. After their responses, an arrow appeared on the chosen figure. In the self-decision condition, figures with arrows were presented for 300 ms and a fixation point was presented for 800 ms. This was followed by a feedback stimulus. In the other-decision condition, the participants had to respond according to the indicated arrow. Each condition consisted of two blocks and the experiment consisted of 4 blocks of 160 trials. The order of the two conditions was counterbalanced between participants.

### EEG recordings

EEGs (bandpass, 0.16–30 Hz; digitized at 500 Hz) were recorded from five electrodes (Fz, C3, Cz, C4, and Pz) according to the international 10/20 system using a digital EEG (Neurofax 1100, Nihon Kohden Corp.). We used Ag/AgCl electrodes and impedance was kept below 10 kΩ. All electrodes were referenced to ear lobes. To detect eye movements and blinks, electro-oculograms (EOGs) were recorded from electrodes lateral to and below the left eye. EEGs were digitized for epochs of 600 ms starting 200 ms prior to the presentation of the stimulus. Individual trials were rejected when EEG voltages were greater than ±50 μV, which is indicative of excessive muscle activity, eye movement, or other artifacts.

Using ERP data, we measured and compared feedback-related negativity (FRN), which are known to be elicited in response to negative stimuli. FRN is elicited at around 250 ms following negative feedback information, such as that reflecting performance error or monetary loss, and is thought to reflect some aspects of self-monitoring processes [11–13]. However, FRNs were hard to analyze because very few trials led to loss feedback in some participants, which may have led to indistinct FRN waveforms in some patients. We thus measured and compared late positive components, which follow FRNs, and are also thought to be sensitive to performance monitoring [14].

We measured the mean amplitude of the late positive component in the time windows 50 ms prior to and subsequent to the peak latency in each grand averaged waveform. In order to examine the neural activity of self-reference processing, we subtracted the ERP waveform obtained during the other-decision condition from that obtained during the self-decision condition. We measured the mean amplitudes of positive deflection in the subtracted waveforms.

In addition to ERP analysis, we analyzed event-related desynchronization (ERD) and event-related synchronization (ERS) responses to feedback stimuli. ERS and ERD indices are calculated using EEG data and reflect different aspects of cognitive processes not usually reflected by ERPs. Concerns regarding task-induced gamma oscillations (frequencies over 30 Hz) has been an issue in schizophrenia research (for a recent review, see [15]). We measured ERS and ERD in the same manner as task-induced gamma activity is measured. We performed on-time frequency analysis to evaluate ERD and ERS following the feedback stimulus using an open-source toolbox running under the MATLAB (MathWorks) environment [16]. The time window to calculate ERS and ERD was an epoch of 800 ms starting 200 ms prior to the presentation of the feedback stimulus.

### ERP data analysis

Three-way repeated-measures analyses of variance (ANOVAs) with Greenhouse-Geisser corrections were performed to compare difference in amplitude of the late positive component. We used group (patients and controls) as the between-subjects factor. The within-subject factors were decision (self-decision and other-decision) and electrode (Fz, C3, Cz, C4, and Pz). We carried out this ANOVA analysis for both gain and loss outcomes. We also performed post-hoc Tukey-Kramer tests. In addition, two-way repeated-measures ANOVAs with Greenhouse-Geisser corrections were performed to compare positive deflections in the subtracted waveforms (self-decision condition minus other-decision condition). We used group (patients and controls) as the between-subjects factor. The within-subject factors were outcome factor (gain and loss) and electrode (Fz, C3, Cz, C4, and Pz).

### Time-frequency analysis

We compared the average power during the pre-stimulus baseline period to that following the feedback stimulus. The frequency axis ranged from 4 to 100 Hz. After collecting the power-change plot for each participant during each condition, we used statistical analysis to detect differences between groups during each condition using EEGLAB. We did not compare the data obtained during the loss and gain conditions, as the trial numbers used in the time frequency analysis were quite variable between the two conditions. We therefore combined the EEG data obtained during the gain and loss conditions.

To determine the significance of the group effect, a two-sample t test between normal control subjects and patients with schizophrenia was performed on each data point in the two corresponding power-change spectra during both conditions (self-decision and other-decision). We performed corrections for multiple comparisons, and a cluster-based, nonparametric randomization test [17]. We followed the same procedure as that used by Dube et al. [18], who had a statistical design similar to ours. The cluster-based approach was used to compare power change during the two conditions at every electrode. In this analysis, time-frequency points at which the t value exceeded the .05 level were clustered via spatial adjacency. The sum of the t values obtained from the cluster with the maximum sum was used as the test statistic. In order to avoid the problem of multiple comparisons, a reference distribution of test statistics was generated by randomly permuting the data across the two conditions 1,000 times. Permutation statistics determined the number of contiguous points that would be expected by chance (the null distribution). Therefore, a cluster was considered significant if the number of time-frequency points was greater than that expected by chance.

Frequency bands were defined as follows: theta = 4–8 Hz, alpha = 8–14 Hz, beta = 14–30 Hz, and gamma 30–100 Hz. The beta band is further subdivided in a lower-range beta band (14–18 Hz) and an upper-range beta band (18–30 Hz) [19]. Similarly, the gamma band (30–100 Hz) includes the low-gamma (30–80 Hz) and a part of the high-gamma (80–160 Hz) range [20].

## Results

### Behavioral measures

**Fig 2:**
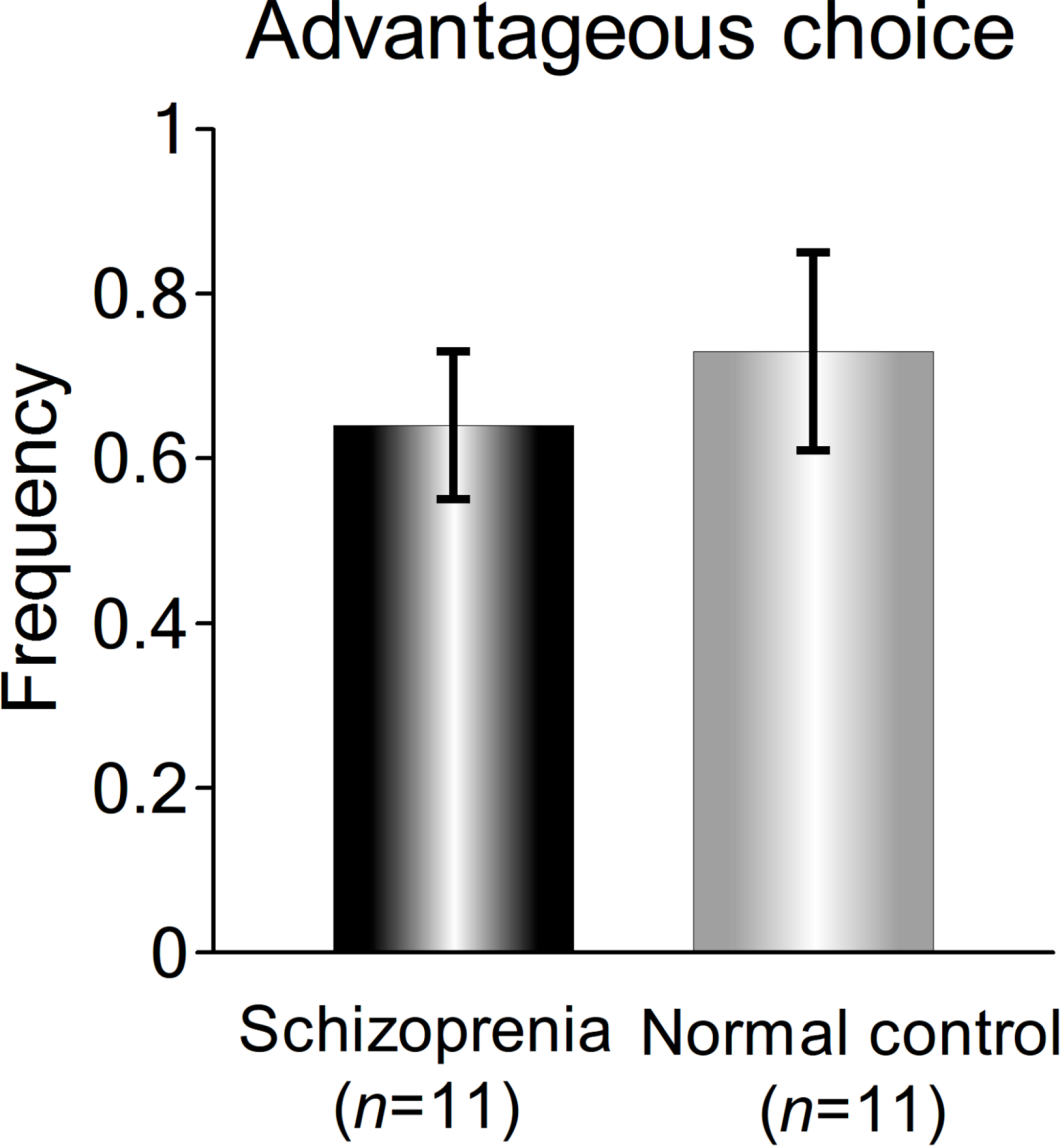
Frequencies of choices for advantageous options in the self-decision condition. There was no statistically significant difference in the choice of the advantageous option between normal controls and patients with schizophrenia (P > 0.05).

During the self-decision condition, the participants had to choose one of the figures, but did not have to do so in other-decision condition. We measured preferences for the two choices in the self-decision condition and compared them between normal control subjects and patients with schizophrenia. Fig 2 shows the numbers of choices for each option. The mean rates of selection of the disadvantageous choice (the left figure, assigned a value of “-1” with a probability of 0.8) were 0.27 (SD = 0.12) in normal subjects and 0.35 (SD = 0.08) in patients with schizophrenia. The mean rates of selection of the advantageous choice (the right figure, assigned a value of “+1” with a probability of 0.8) were 0.73 (SD = 0.12) in normal subjects and 0.64 (SD = 0.09) in patients with schizophrenia. We performed a Student's t-test on the rate of selection of the advantageous choice between normal subjects and the patients. There was no group difference in the preference for the advantageous choice (p > 0.05). This result indicates that learning ability and/or motivational processes to explore the rule and increase one’s points as much as possible did not differ between normal controls and patients with schizophrenia.

### Analysis of the amplitudes of late positive components of ERPs in response to feedback stimuli

**Fig 3:**
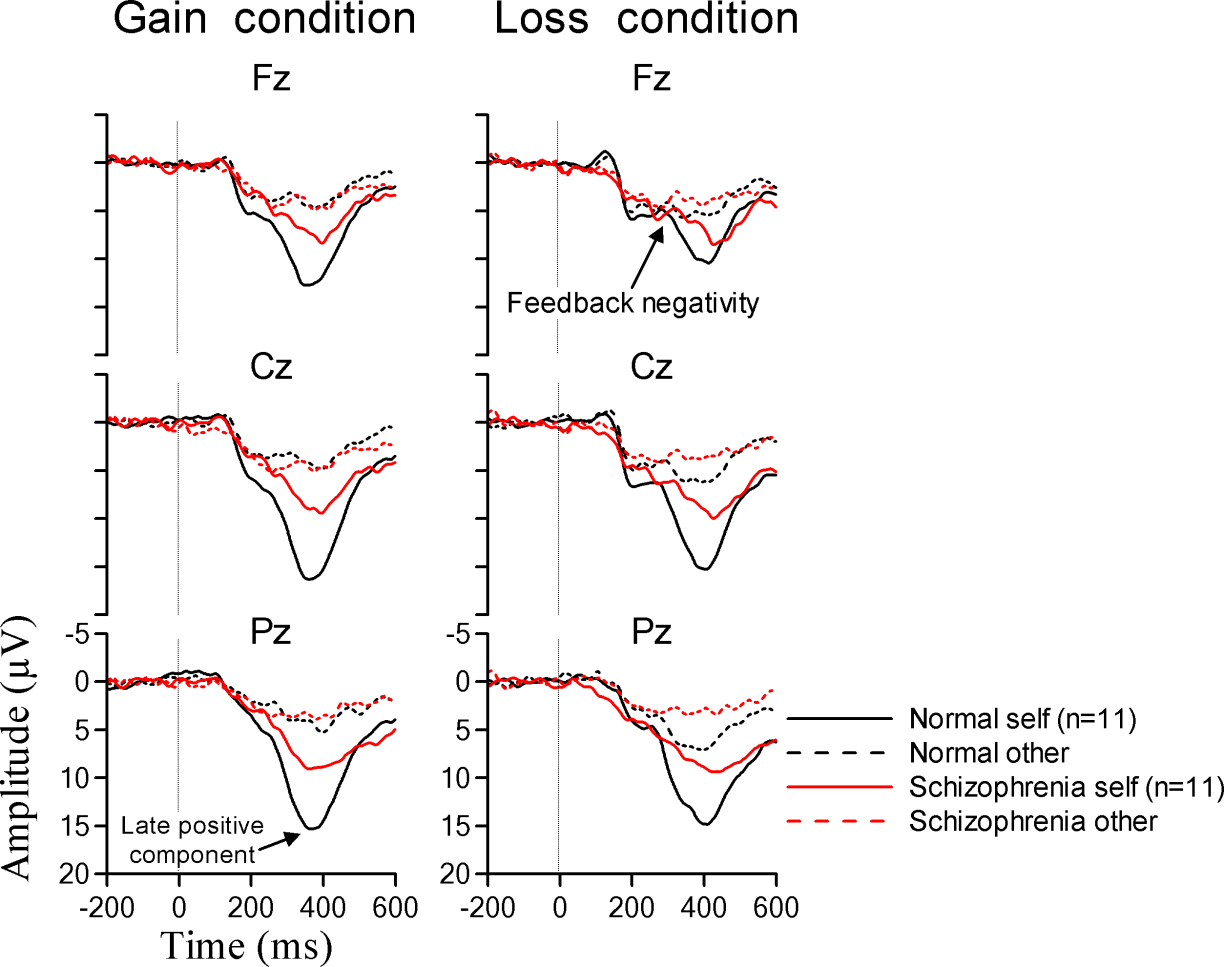
Grand-averaged waveforms in response to feedback stimuli. In the present study, we analyzed the mean amplitudes of positive deflections with peaks at around 400 ms. There were significant interactions between the decision and group factors for both gain and loss outcomes. Subsequent analysis indicated that the mean amplitude of the late positive component in the schizophrenia group was decreased more than it was in the normal subjects, but only in the self-decision condition.

Fig 3 shows grand-averaged waveforms for each condition and group. Based on an inspection of the individual averaged waveforms, we could identify a positive deflection at approximately 350 ms in all conditions. There were main effects of decision in trials that led to both gain and loss outcomes (gain outcome, F (79.0), p < 0.001; loss outcome, F (114.2), p < 0.001). The mean amplitudes of the late positive components during the self-decision condition for both gain and loss outcomes were larger than those during the other-decision condition in both groups. In addition, there was a significant interaction between the decision and group factors in trials leading to both gain and loss outcomes (gain outcome, F (12.3), p = 0.002; loss outcome, F (6.8), p = 0.017). Since we observed a significant interaction between the decision and group factors, we performed ANOVAs with the between-subjects factor group (patients and controls) on the self and other conditions. There was a significant main effect of group in the self-decision condition for both gain and loss outcomes (gain outcome, F (11.1), p = 0.003; loss outcome, F (8.3), p = 0.009). No significant effects were found in the other-decision condition for either gain or loss outcomes (gain outcome, F (0.9), p = 0.33; loss outcome, F (0.003), p = 0.96). This indicates that the mean amplitude of the late positive component in the schizophrenia group was decreased more than that for the normal subjects only in the self-decision condition.

**Fig 4:**
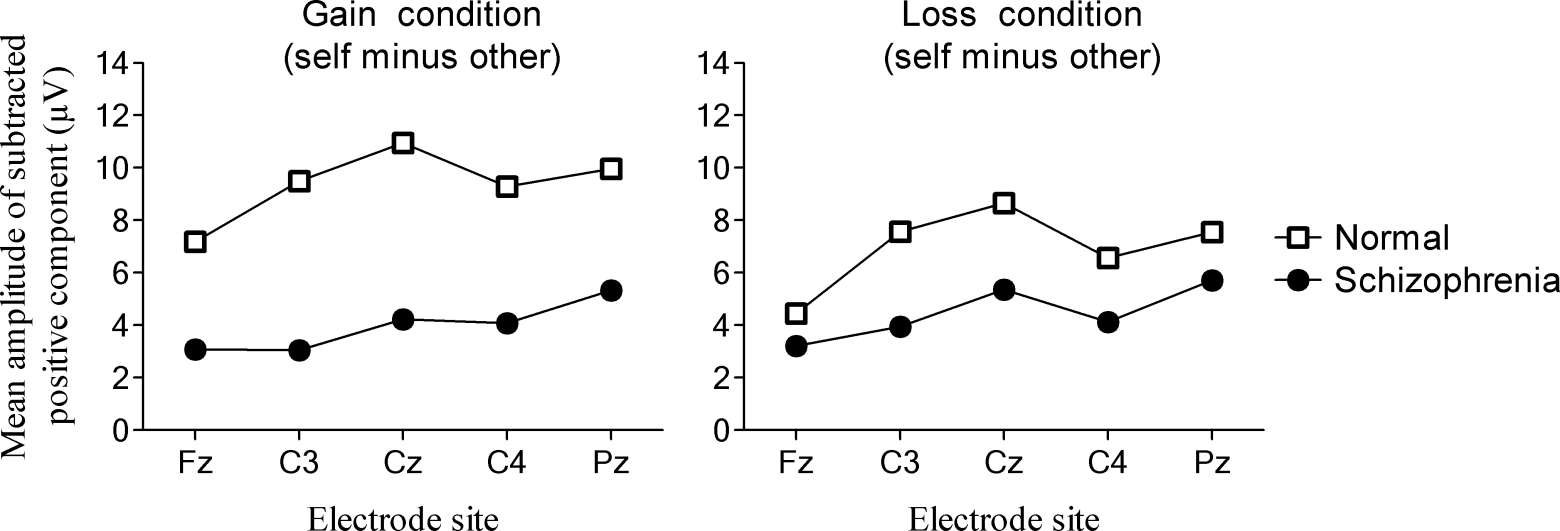
Mean amplitude of the positive deflection in subtracted waveform (self-decision condition minus other-decision condition). This positive deflection reflected the neural activity associated with the self-reference effect. The mean amplitude of the positive deflection in normal subjects was larger than that in patients with schizophrenia for both gain and loss outcomes.

Fig 4 shows the mean amplitudes of the positive deflections in subtracted waveform (self-decision condition minus other-decision condition). The mean amplitude of the positive deflection in subtracted waveform reflects the neural activity associated with the self-reference effect. There was a significant main effect of group (F (12.6), p = 0.002). The mean amplitude of the positive deflection in normal subjects was larger than that in patients with schizophrenia for both gain and loss outcomes.

### Comparison of power-spectrum changes in response to feedback stimuli

**Fig 5:**
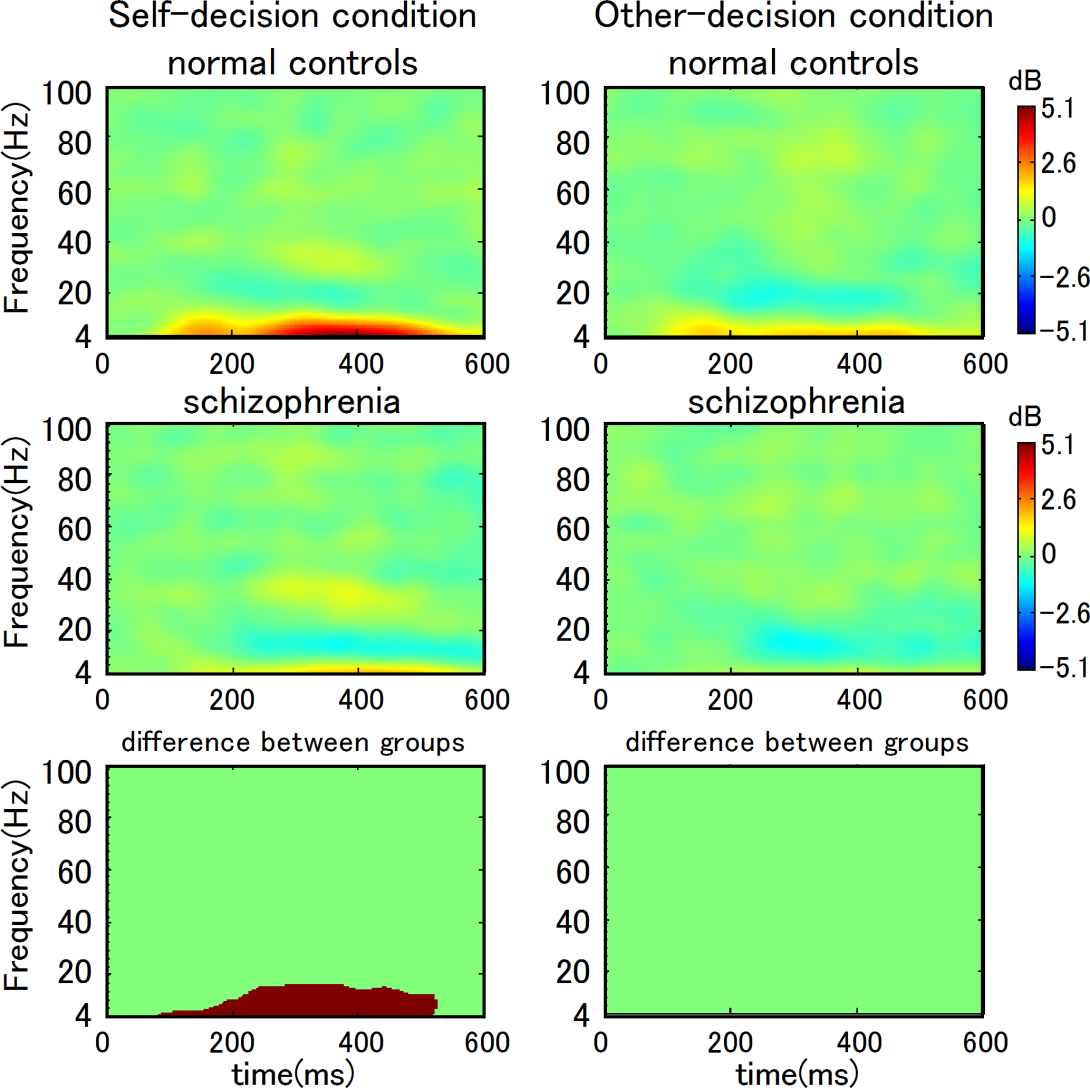
Time-frequency pattern and statistically significant differences in feedback information at Cz. We observed a significant increase in theta and alpha activity (4–13 Hz) during the 100–500 ms after the feedback in the self-decision condition in normal controls when compared to patients with schizophrenia (lower left). In the other-decision condition, there were no significant differences between groups (lower right).

We compared power-spectrum changes in response to feedback stimuli between patients with schizophrenia and normal controls in each condition. Fig 5 shows the time-frequency patterns and points with statistically significant responses to feedback information at Cz. There were significant differences in the self-decision condition at the central scalp area (C3, Cz, and C4). We observed significantly larger increases in theta and alpha spectral power (4–13 Hz) in normal controls than in patients with schizophrenia in the self-decision condition. We observed particularly significant increases in these bands 100–500 ms after the feedback stimulus at the C3, Cz, and C4 electrodes. We did not observe statistically significant changes in the other-decision condition.

## Discussion

The purpose of this study was to investigate neural responses to changes in external events introduced by self- and other-decision-making. We compared the ERP waveforms and power-spectrum changes associated with the self-reference effect between patients with schizophrenia and normal subjects. The ERPs in normal subjects were obviously different in the self and other decision conditions. A larger late positive component was elicited in the self-decision condition that in the other decision condition. ERPs in patients with schizophrenia had significantly smaller positive components in the self-decision condition. In addition, the positive deflections in subtracted waveform (self minus other decision condition) were smaller in patients with schizophrenia. Time-frequency analysis indicated that theta and alpha frequency neural oscillations were enhanced by self-decision-making in normal control subjects.

The larger positive deflection observed in the self-decision condition may reflect the self-reference effect provided during self-decision-making. Although almost all previous studies have focused on sensory processing immediately after motor action, we examined the neural responses of cognitive appraisal processes to an external event (feedback information) about 800 ms after the motor action of the button press. Due to it temporal separation, this larger positive deflection at the time of feedback stimulus onset does not reflect the efference copy (corollary discharge) generated by the operation of the motor command. Rather, it is quite likely that this neural activity, which is specific to self-decision-making, reflects some aspect of cognitive appraisal based on the self-monitoring associated with the attribution of action or source monitoring. Attribution of action is a process through which the consequences of actions are correctly attributed to the self on the basis of visual inspection of the action. Indeed, several studies have shown that there is misattribution of action following visual feedback specifically in schizophrenia (e.g., [21]). Our results regarding the observation of external events providing visual feedback are similar to those of previous studies. Source monitoring is a memory process that involves judgments regarding the origin or source of information. The source of information comprises the spatial, temporal, and contextual characteristics of an event, as well as the sensory modalities through which it is perceived. Errors of evaluation in source monitoring in schizophrenia have also been reported in several previous studies (e.g., [22]). Therefore, the processes underlying source monitoring and the person who chooses the option and provides feedback regarding the outcome must accompany the evaluation at the onset of every feedback stimulus. As mentioned above, the attribution of action and source monitoring might have some characteristics in common with the self-reference effect observed in the present study. However, we did not manipulate the difficulty of attribution or discrimination of the person who made the decision. As a result, the participant must have been able to attribute the feedback of the outcome to himself or to the assistant explicitly. Although the task design of the present study was not behaviorally difficult enough to induce such errors, we believe that the large positive deflection produced by self-decision-making reflects automatic processes involved in the attribution or monitoring of causality accompanying the evaluation of feedback and impairment specific to schizophrenia.

Another difference in the cognitive processes involved in feedback information between the self and other decision condition involves the differences in the probabilities of gain or loss underpinning the two options (left or right figure). In the other-decision condition, the participants were not able to select the option, and the probability of gain or loss was decided at random. As a result, frequency of gain and loss outcomes was different between self- and other- decision conditions. These differences suggest distinct reward learning processes between two conditions. Firstly it should be noted that behavioral data in self-decision condition were not different in both group. Although many previous studies show that patients with schizophrenia have difficulty of reward learning, patients in present study properly learned and selected the advantageous option. It is unlikely that reduction of late positive component in patients with schizophrenia is associated reward learning processes. Secondly in the debriefing following the experiment, all participants, including patients with schizophrenia, said that they had tried to learn the rule regarding which option was advantageous in each trial in the other-decision condition. Some participants believed that they had found this rule through observation. Therefore, the cognitive processes underlying rule investigation did not differ critically between the two conditions, although the rule was not existent in the other-decision condition. Thirdly previous findings suggest that the late positive component (termed P300) elicited by the feedback stimulus is larger in response to unexpected outcomes and is manipulated with learning of the task rule [23]. This indicates that the late positive component is sensitive to unpredicted feedback information during the learning phase. This is not consistent with our finding of the small positive deflection in the other-decision condition. If the late positive component was only modulated by the rule and the frequencies of the gain or loss outcomes, its amplitude would be larger in the other-decision condition, where feedback is presented randomly. It is thus reasonable to suppose that the self-reference effect affects ERP waveforms in response to feedback stimuli more dynamically.

We should mention the effect of the antipsychotic drug on ERP waveforms in patients with schizophrenia. The late positive component in the present study was closely similar to P300 component in morphology. We thought that neural processing underpinning of P300 component in oddball paradigm has common processes for evaluation of feedback stimuli in our paradigm. In the present study, all patients were administered atypical antipsychotics. Atypical antipsychotics have activator action, unlike conventional typical antipsychotics. A Meta-analysis of P300 indicated that typical antipsychotics do not affect P300 amplitude [24]. In addition, several previous studies which examined the effect of atypical antipsychotics on P300 component demonstrated a significant increase in P300 amplitude [25, 26].Given doses of atypical antipsychotics in the present study were relatively low and thus explicit sedative action was not observed in all patients. Hence we argue it is unlikely that antipsychotics cause the reduction of the late positive component in the self-decision condition in patients.

Time frequency analysis in the present study indicated an increase in theta and alpha activity in normal controls when compared to patients with schizophrenia in the self-decision condition. In general, event-related theta and alpha synchronizations have been thought to reflect several aspects of working memory processing [27]. In the self-decision condition in the present study, the feedback stimulus only provided information regarding the gain or loss of reward. As a result, the cognitive load associated with working memory was very small. The enhancement of theta and alpha rhythms during the feedback stimulus in normal controls is unlikely to reflect working memory processes. To our knowledge, there have been no studies to investigate event-related synchronization in a task similar to our multiplayer gambling task. Neural synchronization reflected by gamma band frequency has been a topic of growing interest in studies of the pathophysiological mechanisms underlying negative symptoms and cognitive dysfunction in schizophrenia [15, 28]. Reductions in task-related gamma activity have been reported in various cognitive processes, including working memory, selective attention, language processing, and social cognition. However, our data-driven statistical analysis of task-related neural oscillation did not find any significant differences in gamma-band frequency. In addition, previous studies of functional implications of task-related theta- and alpha-band frequencies in schizophrenia have been inconclusive.

The larger theta and alpha activity in normal controls reflects the self-reference effect, which is associated with some aspects of the attribution of action and source monitoring, as mentioned above. Our findings that there was no increases in these bands in patients with schizophrenia in the self-decision condition indicates that theta and alpha synchronization accompanies attribution processing to self after one’s own decision-making, and that these processes are disrupted in schizophrenia. Further studies are required to confirm the functional significance of theta and alpha synchronization.

Our study had some limitations. First, the sample size was small. As a result, we could not examine the correlation between the severity of symptoms and ERP amplitude in each condition. It is as yet unclear how the large reduction in the late positive component in patients with schizophrenia in the self-decision condition might be causally related to positive symptoms. Further studies with large samples are required to address the causal relationship between the neurophysiological abnormalities observed in the present study and various clinical indices. In addition, further experiments are required to examine differences among individuals with schizophrenia of different clinical stages. Second, we measured EEG from only five electrode sites. We were thus unable to further analyze the source localization of the late positive components in the self-decision condition. For example, the current source density of the positive component between the self and other decision condition or between normal controls and patients with schizophrenia might be very interesting. Further studies measuring multi-channel EEG and analyzing source activity should be carried out.

## Supporting information

S1 Data This file contains data of grand averaged wave and subtracted positive component, and choice behavior in normal controls and patients with schizophrenia. (XLSX)
